# The Preserving Effect of a Lemon Essential Oil‒Rutin‒Chitosan Composite Coating on Seasoned White Snakehead Fillets and Dynamic Changes in Metabolites

**DOI:** 10.3390/foods15071184

**Published:** 2026-04-01

**Authors:** Jiaxin Han, Tianpeng Chen, Xiaolei Jiao, Qiaolan Zhu, Xinhui Wang, Fanbing Meng, Bingliang Liu, Weijun Chen

**Affiliations:** 1 Meat Processing Key Laboratory of Sichuan Province, College of Food and Biological Engineering, Food Security Publicity and Education Base of Sichuan Province, Chengdu University, Chengdu 610106, China; 15877423632@163.com (J.H.); 202310513309@cdu.edu.cn (T.C.); 212022095135178@cdu.edu.cn (Q.Z.); wangxinhui@cdu.edu.cn (X.W.); mfb1020@163.com (F.M.); liubingliang@cdu.edu.cn (B.L.); 2 Sichuan Neijiang Academy of Agricultural Sciences, Neijiang 641099, China; jiao401lei@126.com

**Keywords:** composite coating, seasoned white snakehead fillets, nontargeted metabolomics, refrigerated preservation

## Abstract

In this study, we investigated the preservation efficacy of a lemon essential oil-rutin-chitosan (CS-LEO/NE-R) composite coating on seasoned white snakehead fillets and systematically evaluated dynamic metabolite changes via untargeted metabolomics. The results demonstrated that the composite coating significantly (*p* < 0.05) suppressed microbial proliferation while delaying increases in pH, total volatile basic nitrogen (TVB-N), and thiobarbituric acid reactive substances (TBARS), thereby extending the microbial spoilage threshold by 5 days. Untargeted metabolomics identified 2271 metabolites, with differentially abundant metabolites predominantly involving amino acids and their derivatives, organic acids and their derivatives, and glycerophospholipids. KEGG enrichment analysis suggested that the composite coating maintained cellular membrane stability and was associated with alterations in glycerophospholipid, arachidonic acid, and linoleic acid metabolism pathways linked to membrane integrity, enhanced antioxidant defences, and regulation of energy metabolism homeostasis. Concurrent enrichment of α-linolenic acid metabolism further pointed to altered fatty acid metabolism, consistent with reduced lipid peroxidation product accumulation.

## 1. Introduction

*Channa argus* (northern snakehead), taxonomically classified under the order Perciformes, family Channidae, and genus Channa, is a high-value freshwater fish valued for its low intramuscular fat content. It is endemic to southwestern China. A white variety of *Channa argus* discovered in the Jialing River, Sichuan Province, has emerged as a valuable breeding strain containing an abundance of high-quality protein, polyunsaturated fatty acids, and essential trace elements [1]. However, current market sales of white snakehead focus primarily on live fish, with limited processed products. This confines its market reach predominantly to southwestern China, thereby impeding the nationwide promotion of this high-value aquatic resource [2].

With increasing societal development and consumption, demand for convenient, ready-to-eat foods has grown rapidly [3]. In this context, seasoned white snakehead fillets, owing to their processing convenience and high added value, have emerged as a critical development to overcome geographical constraints in live fish sales and expand market reach. However, processed seasoned fillets are subject to accelerated quality deterioration during storage. The high protein and lipid levels in these products increase their susceptibility to enzymatic reactions and oxidative damage during processing and cold-chain distribution, making the development of efficient preservation technologies essential for maintaining product stability [2]. Multiple preservation methods are currently applied to aquatic products, including low-temperature refrigeration, modified atmosphere packaging (MAP), chemical preservatives, and irradiation. Among these, biopreservatives, particularly edible coating technologies based on natural, biodegradable materials, have attracted significant attention for their eco-friendliness and safety.

Chitosan (CS), which is distinguished by its excellent film-forming properties, biocompatibility, broad-spectrum antibacterial activity, and antioxidant capacity, is a promising matrix material for edible coatings [4]. However, studies have shown that single-component CS coatings exhibit insufficient long-term antioxidant protection and mechanical performance for high-lipid fish [5]. To address these limitations, constructing multifunctional composite coatings by integrating natural bioactive compounds has emerged as an effective strategy for enhancing the performance of chitosan-based materials. Lemon essential oil (LEO) possesses potent antibacterial and antioxidant activities as it is abundant in limonene and γ-terpinene [6]. Liu et al. (2023) demonstrated that LEO-loaded ferulic acid-gelatin/chitosan (FG/CS) active coatings effectively improved the storage stability of grass carp fillets [7]. Rutin, a natural flavonoid, can collectively delay lipid oxidation by scavenging free radicals, chelating metal ions, and inhibiting lipoxygenase [8]. In our previous study, we prepared a lemon essential oil-rutin-chitosan (CS-LEO/NE-R) composite coating [6], in which nanoemulsification technology enhanced the stability and bioavailability of active components, resulting in superior antioxidant and antibacterial performance.

In this study, the preservation effect of the CS-LEO/NE-R composite coating on seasoned white snakehead fillets was evaluated. Additionally, an integrated dual-dimensional analysis of physicochemical indices and untargeted metabolomics, with KEGG pathway enrichment, was conducted to elucidate dynamic metabolite changes in fillets during refrigeration following the coating treatment.

## 2. Materials and Methods

### 2.1. Materials

LEO, CS, and rutin were purchased fromShanghai Yuanye Biological Co., Ltd., Shanghai, China. White snakehead fillets were obtained from Shiling Vegetable Market, Chengdu, China. Food-grade composite phosphates were supplied by Xuzhou Hengshi Food Co., Ltd., Xuzhou, China, and cooking wine (Qianhe brand) was procured from a Shiling Haolego supermarket, Chengdu, China.

Methanol (of chromatographic grade) was purchased from Merck KGaA, Darmstadt, Germany; acetonitrile and acetic acid (both of chromatographic grade) were purchased from Shanghai Xingke High Pure Solvent Co., Ltd.,Shanghai, China; and plate count agar was obtained from Beijing Aoboxing Bio-Tech Co., Ltd., Beijing, China.

### 2.2. Preparation of Seasoned White Snakehead Fillets

The fish fillets were cut into 3.0–3.5 mm thick slices. The surface moisture of the slices was blotted dry with sterile kitchen paper before ultrasound-assisted marination. The marinade composition (based on fillet mass) was as follows: 2% (*w*/*w*) composite phosphates and 4% (*v*/*w*) cooking wine, with a solid-to-liquid ratio of 1:3 (g/mL). The ultrasonic treatment parameters were 495 W for 30 min at room temperature.

### 2.3. Preparation of the CS-LEO/NE-R Composite Coating

The coating was prepared according to the report by Han et al. (2025) [6] using the following procedures. A rutin nanoemulsion was prepared by homogenizing rutin (0.3 mg/mL) in 2% (*v*/*v*) Tween-80 solution at 10,000 rpm for 2 min. Lemon essential oil (LEO) was then blended at a 5:95 (*v*/*v*) ratio and re-homogenized (10,000 rpm, 10 min), followed by ultrasonication using a JY92-IIN ultrasonic cell crusher (Ningbo Xinyi Ultrasonic Equipment Co., Ltd.) at 500 W for 8 min (20 °C).

Chitosan (1.5%, *w*/*v*) was dissolved in 1% (*v*/*v*) acetic acid with stirring for 2 h at 25 °C, and glycerol (5%, *w*/*v*) was added as a plasticizer. After filtration, 4% (*v*/*v*) of the prepared emulsion was incorporated into the chitosan solution, and the mixture was homogenized at 12,000 rpm for 4 min to obtain the CS-LEO/NE-R composite coating.

### 2.4. Seasoned White Snakehead Fillet Preservation

The seasoned white snakehead fillets were randomly divided into two groups. The first group of samples was impregnated in 1000 mL of the CS-LEO/NE-R composite coating for 30 s, with a coating solution volume-to-fillet mass ratio of 3:1 (mL/g); then drained, wrapped in polyethylene cling film, and labelled as the experimental group (designated as CS-LEO/NE-R). The samples without composite coating treatment comprised the control group (designated as CK). All samples were placed in polyethylene trays and maintained at 4 ± 0.5 °C.

### 2.5. pH Analysis

Exactly 5.0 g of the grated sample was blended with 50 mL of deionized water and then left to stand for 0.5 h. The supernatant, after passing through filter paper, was measured using a pH metre [9].

### 2.6. Thiobarbituric Acid Reactive Substances (TBARS) Analysis

TBARS analysis was performed according to the method described by Peng et al. (2024) [9]. A mixture of 5 g of sample and 50 mL of trichloroacetic acid solution was homogenized and then shaken at 50 °C for 0.5 h. After cooling and double filtration, 5 mL of filtrate was reacted with 5 mL of thiobarbituric acid solution at 90 °C for 40 min. Absorbance measurements at 532 nm and 600 nm were performed using a BioTek Synergy HTX full-wavelength microplate reader (Agilent Technologies, Inc., Santa Clara, CA, USA), controlled by Gen5 software (version 3.14, Agilent Technologies, Inc., Santa Clara, CA, USA).

### 2.7. Total Volatile Basic Nitrogen (TVB-N) Analysis

TVB-N analysis was conducted in accordance with the Chinese National Standard GB 5009.228-2016 [10]. Briefly, about 5.0 g of the homogenized sample was extracted and then steam-distilled with a suspension of added magnesium oxide (1 g/L). The volatile nitrogenous substances were absorbed in a boric acid absorption solution (20 g/L). Subsequently, the solution was titrated with a 0.01 mol/L hydrochloric acid standard solution. The TVB-N contents were determined from the volume of hydrochloric acid consumed and expressed as mg/100 g.

### 2.8. Total Viable Count (TVC) Analysis

TVC was determined based on the report of Wang et al. (2024) [11]. Briefly, 5 g of the sample was blended in 90 mL of 0.9% sterile saline using a 60 s homogenization step. After serial ten-fold dilutions, three selected dilutions (0.1 mL) were spread onto plate count agar. The plates were incubated at 36 °C for 48 ± 2 h to enumerate colonies. The results are expressed as log CFU/g.

### 2.9. Nontargeted Metabolomics Analysis

After being ground under liquid nitrogen, a sample (20 ± 1 mg) was moved into a centrifuge tube and mixed with 400 μL of ice-cold aqueous methanol (70%, *v*/*v*) containing internal standards. The mixture was vortexed (1500 rpm, 5 min), ice-incubated (15 min), and centrifuged (12,000 rpm, 4 °C, 10 min). Subsequently, 300 μL of supernatant was transferred to fresh tubes, held at −20 °C for 30 min, and recentrifuged (12,000 r/min, 4 °C, 3 min). The final extracts (200 μL of supernatant) were analyzed by UPLC-MS/MS.

Chromatographic separation was performed using a Waters ACQUITY Premier HSS T3 column (1.8 µm, 2.1 mm × 100 mm) at 40 °C, using 0.1% formic acid in water (A) and 0.1% formic acid in acetonitrile (B) as mobile phases. The gradient programme (0.4 mL/min) was the following: 0–2 min, 5–20% B; 2–5 min, 20–60% B; 5–6 min, 60–99% B; 6–7.5 min, 99% B; 7.5–7.6 min, 99–5% B; 7.6–10 min, 5% B. The injection volume was 4 µL. Mass spectrometry operated in dual-ionization mode [ESI+ (3.5 kV), ESI− (−3.2 kV)] with parameters as follows: curtain gas, 30 psi; nebulizer gas, 5 psi; desolvation temperature, 300 °C. Full-scan data were acquired from *m/z* 75 to 1000 with a resolution of 35,000. A pooled quality control (QC) sample was analyzed after every 10 experimental injections to monitor system stability. Metabolites were annotated based on the self-constructed metabolic database, Human Metabolome Database, and METLIN metabolite database.

### 2.10. Statistical Analysis

Raw data were converted (ProteoWizard) and processed (XCMS) for peak detection, alignment, and retention time correction. Peaks with a missing rate > 50% within any group were removed; remaining missing values were imputed (using k-nearest neighbour). Data were normalized using support vector regression (SVR). Metabolites were identified against standard databases, retaining features with an identification score > 0.5 and QC coefficient of variation < 0.3.

Three independent biological replicates were performed for each experimental group and time point, with each replicate originating from a distinct fish fillet (considered the experimental unit). Data analysis employed analysis of variance (ANOVA) in SPSS 27.0 (IBM SPSS Inc., Chicago, IL, USA), with a significance threshold of *p* < 0.05. Metabolite annotation referenced the Human Metabolome Database (HMDB v5.0). Differentially abundant metabolites were functionally profiled through KEGG pathway enrichment (release 2023.1) for pathway mapping. Temporal expression trends were analyzed via the STEM (version 1.3.11) clustering algorithm to screen for differentially abundant metabolites with *p* < 0.05. 30 temporal patterns were set up to observe the expression patterns of the differentially abundant metabolites at different times.

## 3. Results and Discussions

### 3.1. pH

**Figure 1:**
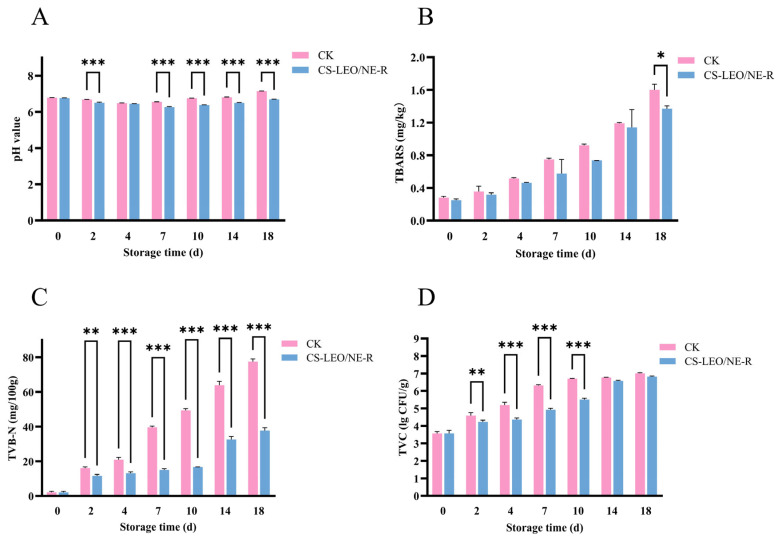
Changes in the physicochemical and microbiological quality indices of seasoned white snakehead fillets during different storage periods. (**A**–**D**) Changes in pH, TBARS, TVB-N, and TVC. * indicates a significant difference at <0.05, ** indicates a significant difference at ≤0.01, *** indicates a significant difference at ≤0.001.

Figure 1A reveals that the pH values of both groups decreased, then increased, and the control group showed a significantly greater increase (*p* < 0.05). The reduction in pH in the initial storage phase may have been due to the decomposition of phosphocreatine, adenosine triphosphate (ATP), and other substances, which released acidic compounds. The pH reduction in the early storage phase was primarily driven by the anaerobic glycolysis of muscle glycogen, which produced lactic acid [12]. On day 14, the pH rose to 6.81 ± 0.02 in the control group but only rebounded to 6.50 ± 0.04 in the treated group. Throughout the storage period, significant differences in pH (*p* < 0.05) were maintained between the two groups. This may have been caused by protein autolysis and the microbial production of basic nitrogenous compounds, such as ammonia [13]. In the later storage stage, the treated group showed a lower pH, indicating that the CS-LEO/NE-R composite coating had an antimicrobial effect. A similar trend was observed by Lin et al. (2025) [14] in rhubarb fish fillets treated with a poly(vinyl alcohol)/chitosan/vanillin nanoparticles (PVA/CS/NP) film.

### 3.2. TBARS

TBARS is an indicator of lipid oxidation in meat. Higher TBARS values indicate more severe lipid oxidation [15]. In fish, TBARS values above 1 mg/kg are generally considered unacceptable. The TBA values of both groups showed an increasing trend over time, but the rate of increase in the treated group was significantly lower (*p* < 0.05) than that in the control group (Figure 1B). The value of the control group rose from an initial 0.273 ± 0.001 mg/kg to 1.194 ± 0.001 mg/kg on the 14th day, with a notably accelerated rate of increase after day 5 (*p* < 0.05). In contrast, the TBA value of the composite coating-treated group showed a relatively slower upward trend. Throughout the storage period, samples treated with the CS-LEO/NE-R composite coating exhibited significantly reduced TBA values (*p* < 0.05) compared to the control group. This result demonstrated that the treatment retarded lipid oxidation. This finding corroborates subsequent metabolomics results and is consistent with a previous report [16].

### 3.3. TVB-N

According to the Chinese National Standard GB 10136-2015, the acceptable TVB-N value of prepared animal aquatic products is <30 mg/100 g. Figure 1C shows that the TVB-N values of both groups increased with storage time. However, the rates of this increase were significantly different (*p* < 0.05). The initial value of the control group was 2.10 ± 0.99 mg/100 g, which rose to 39.90 ± 0.99 and 63.00 ± 1.99 mg/100 g on the 7th and 14th day, respectively. In contrast, the treatment group exhibited a markedly slower rise in TVB-N value, reaching only 32.20 ± 1.99 mg/100 g on the 14th day. This delay in TVB-N accumulation, along with the extension of shelf life (*p* < 0.05), demonstrated the effectiveness of the composite coating in preservation. These results indicate that the CS-LEO/NE-R coating delayed protein degradation and the accumulation of spoilage metabolites during the storage of the seasoned white snakehead fillets [17]. The effectiveness of edible composite coatings in delaying protein degradation and TVB-N increase, as observed in this study, aligns with broader findings in aquatic product preservation research [18].

### 3.4. TVC

Figure 1D shows a progressive rise in TVC values for both groups during storage, with the composite-coating-treated group exhibiting a slower increase rate. The Chinese National Standard GB 10136-2015 stipulates a limit of 5 lg CFU/g of TVC in animal aquatic products. The TVC of the control group reached 5.20 ± 0.11 lg CFU/g on day 4, whereas that of the treated group reached 5.45 ± 0.01 lg CFU/g on day 10. This may have been due to the positive charge of the CS molecules and the negative charge of the microbial membranes, which could inhibit microorganism growth to some extent [19]. In addition, LEO and rutin could also act as antimicrobial agents. Moreover, the composite coating could form a protective film on the sample surfaces, effectively reducing direct contact between them and the air and thus inhibiting the growth of microorganisms. It should be noted that although this study demonstrated the composite coating’s overall antimicrobial efficacy through TVC analysis, the specific spoilage microorganisms affected remain unidentified. These include psychrotrophic bacteria, which are the primary spoilage agents under refrigerated conditions, as well as anaerobic or facultatively anaerobic bacteria (e.g., lactic acid bacteria) whose growth may not be fully captured by current TVC methods. Future investigations should employ selective enumeration of psychrotrophic bacteria, combined with 16S rRNA gene sequencing, to track microbial community dynamics and precisely identify the key bacterial taxa inhibited by the composite coating. This approach would yield deeper ecological insights into the preservation mechanism and provide a more comprehensive understanding of its antimicrobial mode of action.

### 3.5. Nontargeted Metabolomics

To gain deeper insight into the preservation mechanism of the CS-LEO/NE-R composite coating on seasoned white snakehead fillets and to elucidate the dynamic changes in metabolites during storage, nontargeted metabolomics analysis was performed at selected key storage time points based on the trends observed in the physicochemical and microbial indicators described above. As the total viable count (TVC) in the control group had already exceeded the acceptable limit (>5 lg CFU/g) by day 4, and the TVB-N value surpassed the permissible threshold (>30 mg/100 g) by day 7—indicating that the samples had entered the spoilage stage—while the corresponding indicators in the CS-LEO/NE-R group remained within acceptable ranges during the same period, samples from day 0, 2, and 4 were selected for analysis using UHPLC-Q Exactive HF-X mass spectrometry to reveal the metabolic differences at the initial stage of quality differentiation.

#### 3.5.1. Metabolite Classification and Principal Component Analysis

**Figure 2:**
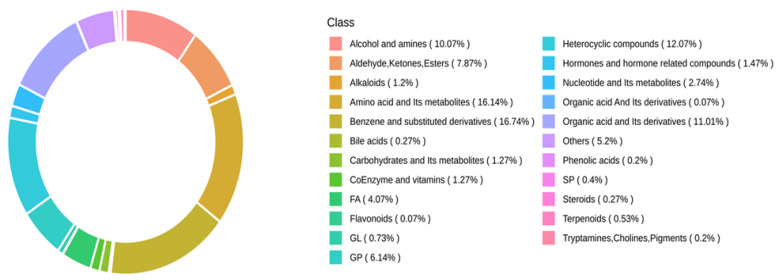
Classification of metabolites.

Figure 2 and Supplementary Table S1 reveal that 2271 metabolites were identified and annotated. The main compound categories of differential metabolites were Benzene and substituted derivatives, Amino acids and their metabolites, Heterocyclic compounds, Organic acids and their derivatives, and Alcohols and amines.

**Figure 3:**
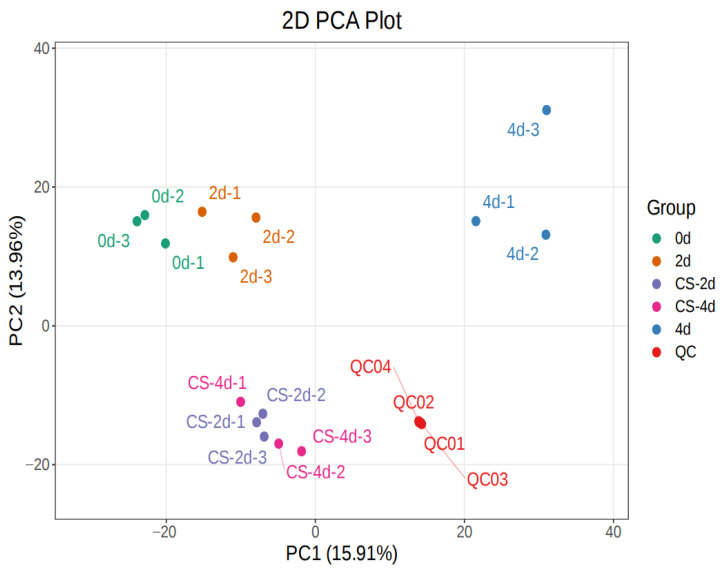
PCA of samples.

**Figure 4:**
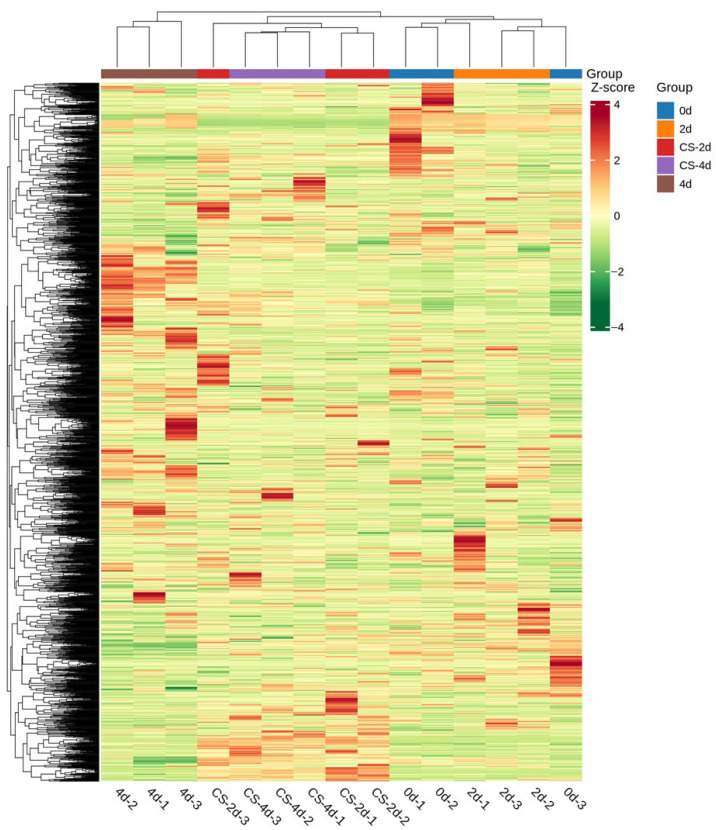
Overall clustering of samples.

In Figure 3, the QC samples are closely distributed near the centre point, indicating that the errors caused by the instrument during sample extraction and data acquisition were small and that the test data are reliable [20]. The three biological replicates (fillet samples) at each time point clustered together with high replicate homogeneity, indicating statistically significant changes in metabolite levels during the preservation of seasoned white snakehead fillets [21]. Hierarchical cluster analysis (HCA) reveals that significant changes occurred in metabolites of the composite coating-treated group during storage, and three biological replicates of seasoned white snakehead fillets from each preservation period cluster together (Figure 4). This suggests that the data are highly reliable and reproducible [21].

#### 3.5.2. Orthogonal Partial Least Squares DISCRIMINANT Analysis (OPLS-DA)

**Figure 5:**
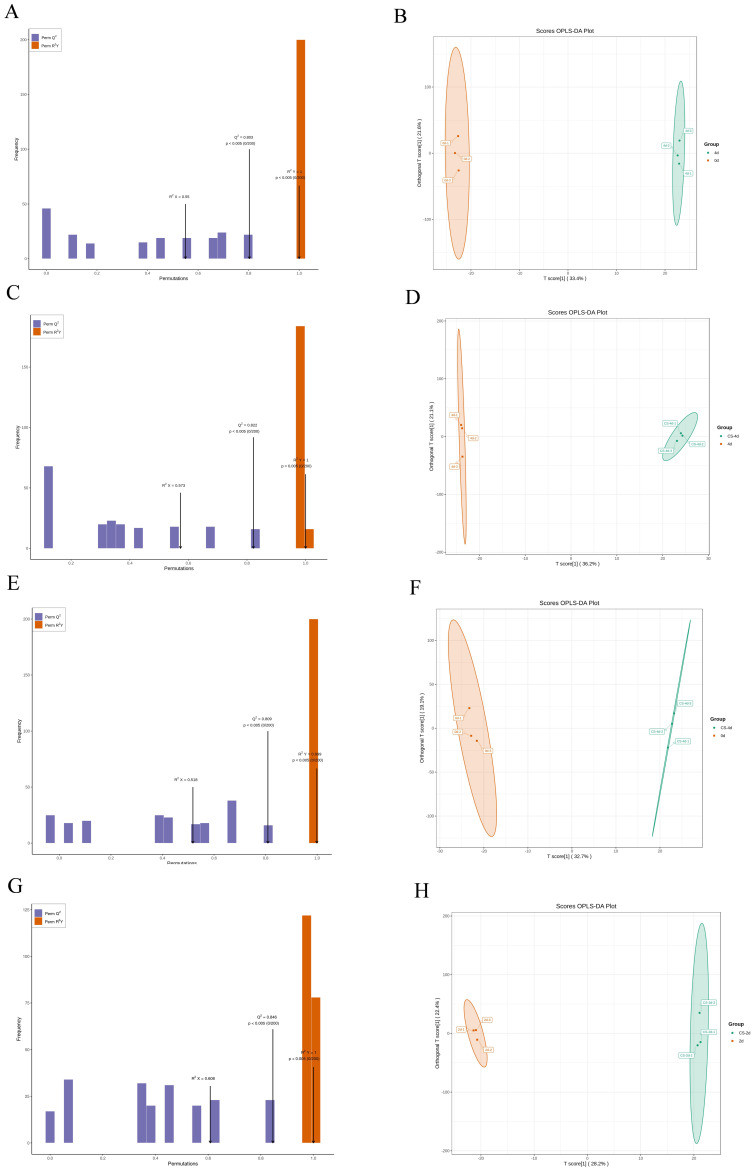
Plots of OPLS-DA scores from two-by-two comparisons for differentially enriched metabolites (**A**,**C**,**E**,**G**) and model validation (**B**,**D**,**F**,**H**). (**A**,**B**): 0 d vs. 4 d. (**C**,**D**): CS-4 d vs. 4 d. (**E**,**F**): CS-4 d vs. 0 d. (**G**,**H**): CS-2 d vs. 2 d.

In the OPLS-DA model, R^2^X and R^2^Y denote the explanatory rates for the X and Y matrices, respectively, and Q^2^ denotes the predictive ability. Q^2^ > 0.5 means that the model can be considered valid, and Q^2^ > 0.9 means that it is excellent; the closer R^2^Y and Q^2^ are to 1, the more stable and reliable the model is [20,22]. Figure 5 shows that the values of the model’s R^2^X range from 0.518 to 0.608, all R^2^Y values are equal to 1, and Q^2^ ranges from 0.803 to 0.846. These results demonstrate the stability and reliability of the model.

#### 3.5.3. Analysis of Significantly Different Enriched Metabolites

**Figure 6:**
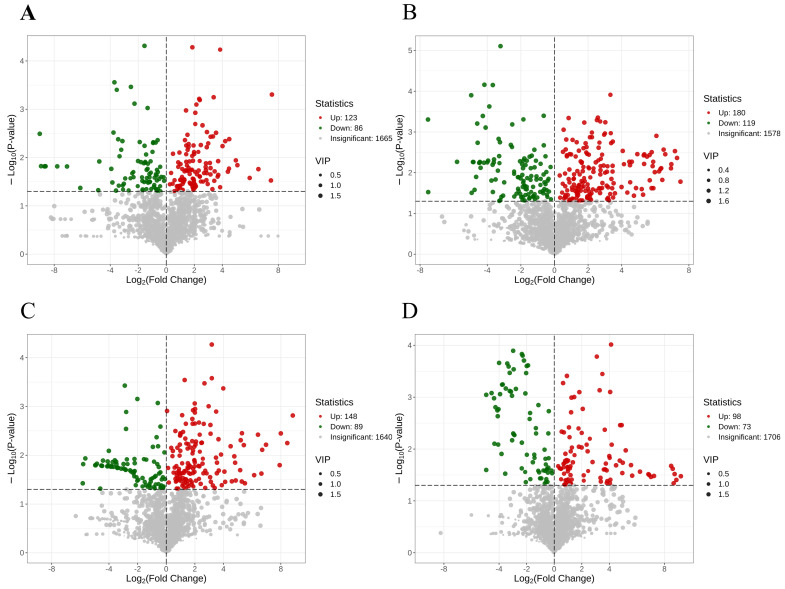
Differential substance metabolism volcano plots for the four comparison groups ((**A**): 0 d vs. 4 d; (**B**): CS−4 d vs. 4 d; (**C**): CS−4 d vs. 0 d; (**D**): CS−2 d vs. 2 d).

Variable importance in projection (VIP) > 1.0 with *p* < 0.05 was used as the screening index for differentially abundant metabolites, and a total of 916 significantly differentially abundant metabolites were identified [23]. Figure 6 displays counts of differential metabolites per comparison group. Significantly upregulated and downregulated metabolites are denoted by red and green dots, respectively. The figure shows that 209 metabolites were significantly differentially regulated in the 0 d and 4 d comparisons, of which 123 and 86 were up- and downregulated, respectively. In the comparison of CS-4 d and 4 d, 299 metabolites exhibited significant differential regulation, with 180 upregulated and 119 downregulated. A comparison of CS-4 d and 0 d revealed that 237 metabolites were significantly differentially expressed, with 148 upregulated and 89 downregulated. In the comparison of CS-2 d and 2 d, 171 metabolites were significantly differentially regulated, including 98 upregulated and 73 downregulated.

#### 3.5.4. Chordal Analysis of Differentially Abundant Metabolites

**Figure 7:**
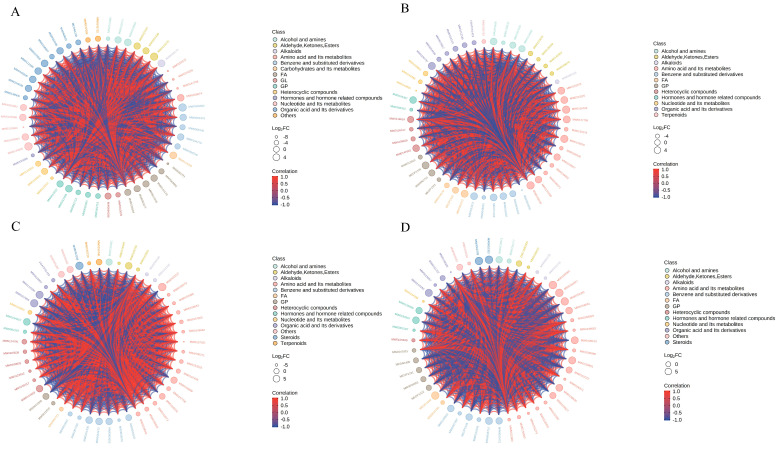
Differentially abundant metabolite chord plots for the four comparison groups ((**A**): 0 d vs. 4 d; (**B**): CS−4 d vs. 4 d; (**C**): CS−4 d vs. 0 d; (**D**): CS−2 d vs. 2 d).

To systematically elucidate the dynamic preservation mechanism of the composite coating treatment, we integrated multi-time-point metabolomic analyses [23]. All differentially abundant metabolites are plotted by default. When the number of differentially abundant metabolites is larger than 50, the top 50 differentially abundant metabolites with the largest VIP are displayed [24,25]. As shown in Figure 7, the changes in differential metabolites primarily included amino acids and their metabolites, benzene and substituted derivatives, and organic acids and their derivatives. The variations in these metabolites played an important role during the coated preservation of seasoned white snakehead fillets [21].

Based on the aforementioned analytical strategy, data nodes with |Log_2_FC| > 4 were selected. A total of 17 significantly different metabolites were identified in the comparison of 0 d with 4 d (Supplementary Table S2), of which 8 were upregulated, and 9 were downregulated. The upregulated metabolites mainly included Pencycuron, Allopurinol riboside, Levulinic acid, and LPC (18:0/0:0), among others; the downregulated metabolites mainly included Inosinic acid, Dihydrogeranylgeranyl diphosphate, Kentsin, and Lys-Gly-Ala-Cys-Lys, among others. The accumulation of Pencycuron, Allopurinol riboside, and Levulinic acid may be associated with microbial secondary metabolite residues, nucleotide metabolism disorders, and exacerbated carbohydrate fermentation, respectively. Their upregulation was consistent with the physicochemical results, which showed that the total viable count in the control group exceeded the acceptable limit (>5 lg CFU/g) by day 4. Specifically, Inosinic acid, an intermediate product of ATP degradation and a flavour compound, was significantly downregulated, reflecting a decline in energy metabolism, which aligned with the trend of pH initially decreasing and then increasing [26]. The accumulation of lysophospholipids, such as LPC (18:0/0:0), was closely associated with the increasing trend in TBARS values, suggesting an exacerbation of lipid oxidation.

In the comparison between CS-4 d and 4 d, 34 metabolites showed significant differences, of which 16 were relatively upregulated, and 18 were relatively downregulated. The upregulated metabolites mainly included peptides (Phe-Thr-Arg-Lys, Val-Pro-Glu-Pro-Lys, etc.), LPC (20:5/0:0), and Kentsin, among others; the downregulated metabolites mainly included Pencycuron, Allopurinol riboside, LPC (0:0/20:3), Deferoxamine, and Phe-Lys-Thr, among others. Analysis revealed that the accumulation of peptide metabolites among the upregulated metabolites may be associated with the inhibition of protein degradation processes, which was consistent with the trend of a lower TVB-N increase rate [27]. The relative upregulation of LPC (20:5/0:0) together with the relative downregulation of LPC (0:0/20:3) suggested a possible rearrangement of phospholipid metabolism, which may be related to membrane structural stability or antioxidant defence mechanisms. Combined with the TBARS data (significantly lower in the treatment group than in the control group), this may be associated with the inhibitory effect of the coating treatment on lipid oxidation, potentially indicating a disruption process of cell membrane structure and suggesting a certain correlation with the degree of spoilage of seasoned white snakehead fillets [28]. In contrast, the relative downregulation of microbial-related metabolites such as Pencycuron and Allopurinol riboside may reflect lower microbial metabolic activity in the treatment group, consistent with the physicochemical results of delayed total viable count growth.

In the comparison between CS-4 d and 0 d, the number of significantly differential metabolites decreased to 24, of which 11 were upregulated, and 13 were downregulated. The upregulated metabolites mainly included Prosapogenin A, Gly-Asp-Ile-Val-Ile, Levulinic acid, and Ser-Pro-Thr, among others; the downregulated metabolites mainly included all-trans-Pentaprenyl diphosphate, Deferoxamine, Phe-Lys-Thr, and Gln-Gln-Asn, among others. Compared with the 0 d vs. 4 d comparison group, the metabolic variation profile in the CS-4 d vs. 0 d group showed distinct characteristics during the same storage period. The sustained upregulation of antimicrobial steroidal metabolites, such as Prosapogenin A, indicated that the antibacterial defence mechanism of the coating treatment remained active after 4 d of storage, consistent with the physicochemical results showing that the total viable count in the treatment group remained consistently lower than that in the control group. The accumulation of oligopeptide metabolites such as Gly-Asp-Ile-Val-Ile and Ser-Pro-Thr may reflect regulation of protein degradation processes, aligning with the trend of a reduced rate of increase in TVB-N [29]. Among the downregulated metabolites, the decrease in terpenoid synthesis precursors such as all-trans-Pentaprenyl diphosphate may suggest an overall reduction in secondary metabolic activity; the downregulation of oligopeptides such as Phe-Lys-Thr and Gln-Gln-Asn was contrary to the peptide degradation trend observed in the control group, indicating that protein degradation processes may have been effectively delayed in the treatment group. Overall, the treatment group exhibited a rebalancing of the metabolite profile after 4 d of storage, consistent with the trends of reduced total viable count and increased TVB-N during the same period.

In the comparison between CS-2 d and 2 d, 25 metabolites were identified as significantly differentially abundant. Among them, 19 were relatively upregulated, and 6 were relatively downregulated. The upregulated metabolites mainly included Prosapogenin A, Digitoxin, Carnitine C12-OH, Leu-Ile-Pro-Leu-Leu, His-Asn-Gly, Salicylic acid, and Ser-Ala-Lys-Lys, among others; the downregulated metabolites mainly included Gln-Gln-Asn, Phe-Lys-Thr, Deferoxamine, and Dihydrogeranylgeranyl diphosphate, among others. Among the upregulated metabolites, the early significant upregulation of antimicrobial steroidal metabolites such as Prosapogenin A and Digitoxin indicated that the antibacterial defence mechanism of the coating treatment was rapidly activated at the initial stage of storage, which was consistent with the trend that the total viable count growth rate in the treatment group was lower than that in the control group. Carnitine C12-OH, as a lipid metabolism-related compound, may be associated with the regulation of fatty acid β-oxidation pathways, suggesting a possible link to the maintenance of energy metabolism. The accumulation of various oligopeptide metabolites such as Leu-Ile-Pro-Leu-Leu, His-Asn-Gly, and Ser-Ala-Lys-Lys may be related to muscle protein degradation caused by endogenous protease activation, and the released short peptides may inhibit lipid oxidation through chelating metal ions [28]. The upregulation of Salicylic acid, a plant-derived anti-inflammatory component, may reduce the production of pro-inflammatory factors by inhibiting the lipoxygenase (LOX) pathway [30]. This finding was consistent with the macroscopic observation that the TBARS value in the composite coating treatment group exhibited a gentle increasing trend and remained significantly lower than that in the control group throughout storage (*p* < 0.05), potentially elucidating the intrinsic mechanism by which the CS-LEO/NE-R coating treatment delays lipid oxidation. Among the downregulated metabolites, the downregulation of oligopeptides such as Gln-Gln-Asn and Phe-Lys-Thr may reflect the selective regulation of specific protein degradation fragments.

#### 3.5.5. KEGG Enrichment Analysis

**Figure 8:**
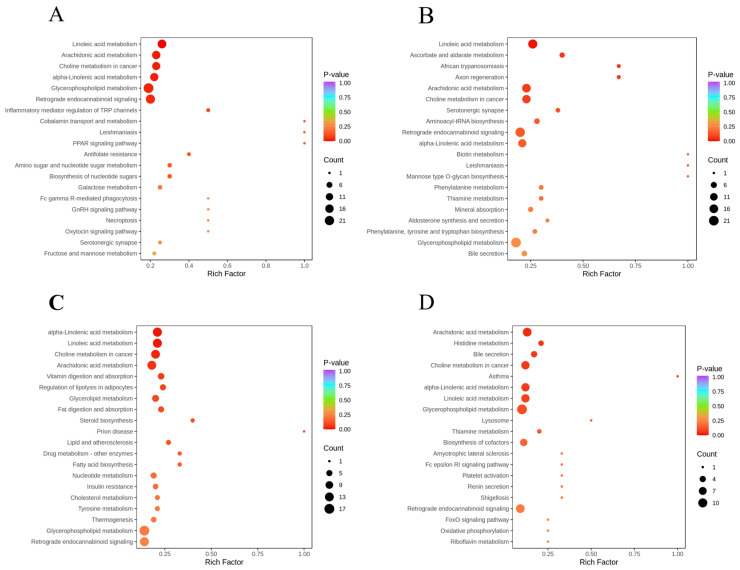
Differentially abundant metabolite pathway enrichment maps for the four comparison groups ((**A**): 0 d vs. 4 d; (**B**): CS-4 d vs. 4 d; (**C**): CS-4 d vs. 0 d; (**D**): CS-2 d vs. 2 d).

Differential metabolites across comparison groups were mapped to the KEGG database to delineate associated metabolic pathways (Supplementary Tables S6–S9), and the results were visualized in bubble plots. Dot scaling reflects the enrichment magnitude of differential metabolites per pathway [12]. The top 20 statistically significant pathways (with *p*-values ranked in ascending order) are presented in Figure 8.

Pathway enrichments analyses revealed that in the comparison of the 0 d and 2 d groups Pathway enrichments analyses revealed that in the comparison of the 0 d and 4 d groups, differentially abundant metabolites were significantly enriched in linoleic acid metabolism, arachidonic acid metabolism, alpha-Linolenic acid metabolism, glycerophospholipid metabolism, choline metabolism in cancer, PPAR signalling pathway and other pathways (Supplementary Table S6). The significant enrichment of linoleic acid, arachidonic acid, and glycerophospholipid metabolism pathways showed strong consistency with the upregulation of lysophospholipids such as LPC (18:0/0:0) and the increasing trend in TBARS values, suggesting that lipid-oxidative degradation pathways were markedly activated during the natural spoilage process [31]. The PPAR signalling pathway, serving as a key regulatory hub for lipid metabolism and inflammatory responses, further corroborated the exacerbation of lipid metabolism disorders and oxidative stress. These findings indicated that, in the control group, after 4 d of storage, pathways related to lipid oxidation and membrane metabolism disruption were widely activated, consistent with the physicochemical results showing that total viable count exceeded the acceptable limit and that TVB-N and TBARS values increased rapidly. Furthermore, the synergistic interaction between choline metabolism in cancer and glycerophospholipid metabolism may help delay cell autolysis and microbial invasion by maintaining the dynamic balance of cellular membrane phospholipids [32].

Between the 4 d and CS-4 d groups, differentially abundant metabolites were primarily enriched in linoleic acid metabolism, arachidonic acid metabolism, alpha-linolenic acid metabolism, choline metabolism in cancer, ascorbate and aldarate metabolism, and aminoacyl-tRNA biosynthesis pathways. Compared with the 0 d vs. 4 d group, lipid oxidation-related pathways (linoleic acid metabolism, arachidonic acid metabolism, alpha-linolenic acid metabolism) remained significantly enriched; however, considering the metabolite trends (upregulation of LPC (20:5/0:0) and downregulation of LPC (0:0/20:3)), it is hypothesized that the coating treatment may have altered the specific pathways of lipid oxidation by regulating phospholipid metabolism rearrangement [33]. The enrichment of the ascorbate and aldarate metabolism pathway suggested that the coating treatment might have activated the antioxidant defence system. The enrichment of the aminoacyl-tRNA biosynthesis pathway may be associated with the maintenance of protein synthesis metabolism. Overall, at the critical spoilage threshold, the coating treatment elicited a distinct pathway response pattern compared with the control group by regulating lipid metabolism, activating antioxidant defence, and maintaining protein synthesis [34].

Between the CS-2 d and 2 d groups, differentially abundant metabolites were significantly enriched in the arachidonic acid, alpha-linolenic acid, linoleic acid, glycerophospholipid, choline metabolism in cancer, histidine, and thiamine metabolism pathways. The enrichment of the arachidonic acid metabolism pathway corresponded with the upregulation of anti-inflammatory mediators, such as salicylic acid, potentially reducing pro-inflammatory factor production by inhibiting the lipoxygenase pathway, consistent with the early trend of a gently increasing TBARS value in the treatment group [35]. The enrichment of the glycerophospholipid metabolism pathway suggested that membrane lipid metabolism was regulated at the early stage of storage. The enrichment of the histidine metabolism pathway may be associated with the regulation of endogenous antioxidant synthesis (e.g., carnosine) [36]. Notably, the choline metabolism in the cancer pathway was persistently enriched across multiple comparison groups, indicating that choline metabolism, as a core node in membrane phospholipid synthesis and degradation, may play a key role in the preservation mechanism of the coating treatment. Overall, the enrichment results of CS-2 d vs. 2 d demonstrated that at the early storage stage, when physicochemical indicators had not yet fully diverged, the coating treatment had already initiated multi-level primary defence responses by regulating inflammation-related pathways, membrane lipid metabolism, and antioxidant-associated pathways.

Further comprehensive analysis of pathway enrichment across the four comparison groups revealed overlapping enrichment of multiple metabolic pathways, indicating their core roles in the coating preservation mechanism. Among these, lipid metabolism-related pathways (linoleic acid metabolism, arachidonic acid metabolism, and glycerophospholipid metabolism) were persistently enriched, suggesting that the coating treatment primarily delayed the quality deterioration of seasoned white snakehead fillets by regulating membrane lipid metabolism and oxidative stability, synergistically with antibacterial defence and energy metabolism maintenance. This systematically elucidated the molecular mechanism by which the coating treatment retarded deterioration in quality.

## 4. Conclusions

In this study, the point at which TVC in the control group exceeded the acceptable limit (day 4) was used as the critical metabolic node to evaluate the preservative effect of the CS-LEO/NE-R composite coating on seasoned white snakehead fillets during cold storage and to elucidate its molecular regulatory mechanism via nontargeted metabolomics. Physicochemical analysis demonstrated that the CS-LEO/NE-R coating significantly delayed increases in pH, TVB-N, TBARS, and TVC during storage. Specifically, this treatment extended the microbial spoilage threshold by 5 days and delayed the time to exceed the TVB-N limit by 3 days, thereby effectively maintaining product quality.

Nontargeted metabolomics analysis identified 2271 metabolites, of which 916 were significantly differential metabolites, primarily belonging to categories such as amino acids and their metabolites, organic acids and their derivatives, and benzene and its substituted derivatives. Comparative analysis of the four groups revealed the following: the 0 d vs. 4 d comparison elucidated the characteristic metabolite profile associated with microbial proliferation, lipid oxidation, and energy depletion during natural spoilage; the 4 d vs. CS-4 d comparison clarified that the coating treatment exerted preservative effects at the critical spoilage threshold through multiple pathways, including inducing the accumulation of peptide metabolites, regulating phospholipid metabolism rearrangement, and inhibiting microbial-related metabolites; the CS-4 d vs. 0 d intra-group analysis confirmed that the treatment group entered a metabolic homeostasis state during the later storage period, characterized by sustained upregulation of antimicrobial steroids and persistent inhibition of spoilage-related metabolites; and the CS-2 d vs. 2 d comparison captured the primary responses of the coating treatment, including rapid activation of antibacterial defence, regulation of lipid oxidation, and modulation of energy metabolism. KEGG enrichment analysis demonstrated that the CS-LEO/NE-R coating treatment primarily exerted its preservative effects by regulating core pathways, including arachidonic acid, linoleic acid, and glycerophospholipid metabolism. These pathways collectively coordinated the maintenance of cell membrane integrity, the oxidative stress response, and the regulation of energy homeostasis, thereby elucidating the molecular mechanism by which the CS-LEO/NE-R composite coating delayed the deterioration in quality of seasoned white snakehead fillets.

## Data Availability

The original contributions presented in this study are included in the article/Supplementary Materials. Further inquiries can be directed to the corresponding author.

## Supplementary Materials

The following supporting information can be downloaded at https://www.mdpi.com/article/10.3390/foods15071184/s1. Table S1: List of total metabolites in seasoned white snakehead fillets; Table S2: List of differential metabolites between 0 d and 4 d; Table S3: List of differential metabolites between CS-4 d and 4 d; Table S4: List of differential metabolites between CS-4 d and 0 d; Table S5: List of differential metabolites between CS-2 d and 2 d; Table S6: List of KEGG enrichment of the significantly differential regulated metabolites for the comparison of 0 d vs. 4 d; Table S7: List of KEGG enrichment of the significantly differential regulated metabolites for the comparison of CS-4 d vs. 4 d; Table S8: List of KEGG enrichment of the significantly differential regulated metabolites for the comparison of CS-4 d vs. 0 d; Table S9: List of KEGG enrichment of the significantly differential regulated metabolites for the comparison of CS-2 d vs. 2 d.
